# Technology and Data Collection in Chronic Disease Epidemiology

**DOI:** 10.5888/pcd12.150400

**Published:** 2015-10-29

**Authors:** James B. Holt

In this issue of *Preventing Chronic Disease*, Moodley et al (1) present the results of a spatial analysis of the locations of advertisements for sugar-sweetened beverages (SSBs) and vendors who sell SSBs in relation to the location of schools in 5 neighborhoods in South Africa. In their article, “Obesogenic Environments: Access to and Advertising of Sugar-Sweetened Beverages in Soweto, South Africa,” the authors used a global positioning system (GPS) and a digital camera to gather data on the locations of SSB advertisements and vendors. Their innovative and low-cost approach could be replicated in any setting, including the United States, where time-sensitive point-location data on environmental exposure are needed but are unavailable through more traditional data-collection sources. In this sense, their approach to gathering data is situated within the broader technological developments of volunteered geographic information, crowdsourced data, and GPS-enabled mobile technology for public health (2–6).

Although the main objective of Moodley et al was to provide a descriptive analysis of the intensity of SSB advertising, their approach to using technology deserves to be highlighted because it may be of great value to public health practitioners. To this end, *Preventing Chronic Disease* readers may find valuable some additional examples of the use of handheld GPS devices or smartphones for data collection for chronic disease epidemiology. Smartphones are GPS-enabled, and photographs taken with smartphone cameras are encoded with a GPS location. Software applications for smartphones that allow photographs to be exported and their location information to be stored on a convenient database include commercial applications such as Collector for ArcGIS (Esri, http://doc.arcgis.com/en/collector/) and open-source free applications such as Ushahidi (www.ushahidi.com/product/ushahidi/).

Many recently published studies illustrate how this technological approach has been used in the field. Braun et al (7) provided a comprehensive review of the use of mobile technology for field data collection among community health workers. Aanensen et al (8) described the development of a system to link smartphones to Web applications for the collection of field data, which can include GPS locations, photographs, videos, and audio. Chunara and colleagues (9) cited examples of mobile technology use for rapid reporting of outbreak information, such as malaria in Cambodia. Patel et al (10) described the development and implementation of a smartphone application to measure the presence of smoking in vehicles, in addition to the presence of adult passengers, child passengers, or both; they also stress the advantages of efficiency and standardization and the ability to transmit data from many remote locations to a centralized website for further analysis. Kanter and colleagues (11) developed, field tested, and evaluated a mobile telephone–based nutrition environment survey in Guatemalan supermarkets, and they noted that the mobile application had equivalent reliability and validity to a paper version of the survey and was also faster to use. King et al (12) reviewed advances in and issues related to using mobile technologies to assess the built environment for the purpose of improving active living and healthy eating. Eyler et al (13), in a presentation of case studies for the assessment of physical activity and the built environment, described the development of an iPad application (named iSOPARC) that enables users to collect and manage data elements for the System for Observing Play and Recreation in Communities (SOPARC), which has been validated and in use since 2004. The goal of the mobile application was to increase the use of SOPARC by making it more accessible to a broader range of end users.

Bethlehem et al (14) discussed a different approach to using digital technology to assess neighborhood obesogenic characteristics; instead of collecting data in the field, they remotely analyzed digital photographs from Google Earth and Google Street View. In both cases, they found that assessments were valid and reliable and could be completed in roughly one-half the time as field-based data collection. One drawback of this approach is that not all potential areas of interest have been imaged for Google Earth and Google Street View, particularly areas where cars are prohibited. Furthermore, the date of image collection is not controlled by the health researcher, so a large study may contain data obtained at different times. Nonetheless, this approach is an interesting area for further development and highlights the innovative ways in which digital data are being used.

Mobile technology is changing rapidly, and so are the innovative applications for using it. Curtis et al (15) collected street-level spatial video data in a Haitian community through the analysis of 4 automobile-mounted digital video cameras. The spatial video was viewed in Google Earth, and environmental attributes of interest (eg, standing water, trash, structural integrity of homes) were manually coded; the resulting data were exported to ArcGIS (Esri) for further spatial analysis. As a sign of applications to come, Igoe et al (16) discussed the feasibility of using smartphones for real-time measurement of ultraviolet A (UVA) radiation and aerosol optical depth, both of which are measures of the physical environment that can affect health. It is not too great a stretch to imagine the near future when our smartphones or wearable technology may be able to measure UVA radiation and provide real-time recommendations for limiting sun exposure.

Moodley et al provide a case study of how a group of researchers with a defined research question for a well-documented public health concern used readily available low-cost technology to create a unique spatial database of environmental exposures. This approach has relevance to many different geographic settings and exposures, including data that may be available from commercial vendors but prohibitively expensive (eg, business and marketing data), as well as exposures that are more ephemeral, such as advertising billboards, where existing data sets may not be current for the period studied. Their data collection methods can be adopted by researchers and communities interested in various chronic disease–related exposures or assets (either harmful or protective), such as alcohol and tobacco advertising, fast-food outlets, and farmers markets. Public health practitioners could either adopt their approach of using a handheld GPS in combination with a digital camera or use similar approaches that are available through ready-made commercial or open-source smartphone applications, as this brief sampling of literature suggests.

## References

[1] Moodley G , Christofides N , Norris SA , Achia T , Besada D , Hofman KJ . Obesogenic environments: access to and advertising of sugar-sweetened beverages in Soweto, South Africa. Prev Chronic Dis 2015:140559. 10.5888/pcd12.140559 PMC4651143 26513442

[2] Goranson C , Thihalolipavan S , di Tada N . VGI and public health: possibilities and pitfalls. In: Crowdsourcing geographic knowledge. Sui D, Elwood S, Goodchild M, editors. New York (NY): Springer; 2013. p. 329–40.

[3] Kamel Boulos MN , Resch B , Crowley DN , Breslin JG , Sohn G , Burtner R , Crowdsourcing, citizen sensing and sensor web technologies for public and environmental health surveillance and crisis management: trends, OGC standards and application examples. Int J Health Geogr 2011;10(1):67. 10.1186/1476-072X-10-67 22188675 PMC3271966

[4] Freifeld CC , Chunara R , Mekaru SR , Chan EH , Kass-Hout T , Ayala Iacucci A , Participatory epidemiology: use of mobile phones for community-based health reporting. PLoS Med 2010;7(12):e1000376. 10.1371/journal.pmed.1000376 21151888 PMC2998443

[5] Kerr J , Duncan S , Schipperijn J . Using global positioning systems in health research: a practical approach to data collection and processing. Am J Prev Med 2011;41(5):532–40. 10.1016/j.amepre.2011.07.017 22011426

[6] Stevens KB , Pfeiffer DU . Sources of spatial animal and human health data: casting the net wide to deal more effectively with increasingly complex disease problems. Spat Spatio-Temporal Epidemiol 2015;13:15–29. 10.1016/j.sste.2015.04.003 26046634 PMC7102771

[7] Braun R , Catalani C , Wimbush J , Israelski D . Community health workers and mobile technology: a systematic review of the literature. PLoS One 2013;8(6):e65772. 10.1371/journal.pone.0065772 23776544 PMC3680423

[8] Aanensen DM , Huntley DM , Feil EJ , al-Own F , Spratt BG . EpiCollect: linking smartphones to web applications for epidemiology, ecology and community data collection. PLoS One 2009;4(9):e6968. 10.1371/journal.pone.0006968 19756138 PMC2735776

[9] Chunara R , Freifeld CC , Brownstein JS . New technologies for reporting real-time emergent infections. Parasitology 2012;139(14):1843–51. 10.1017/S0031182012000923 22894823 PMC3760201

[10] Patel V , Nowostawski M , Thomson G , Wilson N , Medlin H . Developing a smartphone ‘app’ for public health research: the example of measuring observed smoking in vehicles. J Epidemiol Community Health 2013;67(5):446–52. 10.1136/jech-2012-201774 23443959

[11] Kanter R , Alvey J , Fuentes D . A novel mobile phone application to assess nutrition environment measures in low- and middle-income countries. Food Nutr Bull 2014;35(3):296–300. 25902589 10.1177/156482651403500302

[12] King AC , Glanz K , Patrick K . Technologies to measure and modify physical activity and eating environments. Am J Prev Med 2015;48(5):630–8. 10.1016/j.amepre.2014.10.005 25891063 PMC7155784

[13] Eyler AA , Blanck HM , Gittelsohn J , Karpyn A , McKenzie TL , Partington S , Physical activity and food environment assessments: implications for practice. Am J Prev Med 2015;48(5):639–45. 10.1016/j.amepre.2014.10.008 25891064

[14] Bethlehem JR , Mackenbach JD , Ben-Rebah M , Compernolle S , Glonti K , Bárdos H , The SPOTLIGHT virtual audit tool: a valid and reliable tool to assess obesogenic characteristics of the built environment. Int J Health Geogr 2014;13(1):52. 10.1186/1476-072X-13-52 25515179 PMC4279584

[15] Curtis A , Blackburn JK , Widmer JM , Morris JG Jr . A ubiquitous method for street scale spatial data collection and analysis in challenging urban environments: mapping health risks using spatial video in Haiti. Int J Health Geogr 2013;12(1):21. 10.1186/1476-072X-12-21 23587358 PMC3685559

[16] Igoe DP , Parisi A , Carter B . Smartphone-based android app for determining UVA aerosol optical depth and direct solar irradiances. Photochem Photobiol 2014;90(1):233–7. 10.1111/php.12185 24117514

